# Heme oxygenase-1 activation inhibits XMRV pathogenesis and carcinogenesis in prostate cancer cells

**DOI:** 10.1186/1742-4690-8-S1-A218

**Published:** 2011-06-06

**Authors:** Subhash Dhawan

**Affiliations:** 1 Laboratory of Molecular Virology, Division of Emerging and Transfusion Transmitted Diseases, Center for Biologics Evaluation and Research, Food and Drug Administration, Bethesda, MD, USA

## 

Heme oxygenase-1 (HO-1) induction by hemin, the active ingredient of an FDA-approved biologic for the treatment of acute porphyries, has been previously shown in our laboratory to effectively inhibit HIV-1 replication. The present study was undertaken to expand these observations to examine the inhibitory role of HO-1 in the pathogenesis of XMRV infection. Hemin-induced HO-1 activation in LNCaP, a prostate cancer cell line susceptible to XMRV infection, markedly down-modulated the cell surface expression of XMRV receptor Xpr1, and significantly inhibited their ability to support productive virus replication. Hemin treatment of XMRV-integrated prostate carcinoma cells 22Rv1 was relatively less effective in suppressing the rapidly replicating XMRV in these aggressive malignant cells; yet it efficiently inhibited XMRV infection of LNCaP cells by about 80% when cultured for three days in the virus-containing 50% conditioned media from 22Rv1 cells. Additionally, HO-1 induction retarded the growth of uninfected LNCaP cells, XMRV-infected LNCaP cells and 22Rv1 cells, and significantly reduced their invasiveness to the reconstituted basement membrane matrix Matrigel, consistent with the lower levels of basement membrane degrading enzyme matrix metalloproteinase-9. These findings indicate a pivotal role of HO-1 as a host cell defense mechanism in prostate carcinogenesis in vitro, and may offer HO-1 induction as a potentially novel therapeutic strategy to control the pathogenesis of XMRV infection.

